# Exploration of the Mechanisms of *Acorus tatarinowii* in the Treatment of Major Depressive Disorder Based on Network Pharmacology and Molecular Docking Techniques

**DOI:** 10.3390/cimb47050342

**Published:** 2025-05-09

**Authors:** Li Han, Siwen Wei, Rong Wang, Yiran Liu, Yi Zhong, Huaiqing Luo

**Affiliations:** 1 Hunan Provincial University Key Laboratory of the Fundamental and Clinical Research on Functional Nucleic Acid, Changsha Medical University, Changsha 410219, China; td20230611@hunnu.edu.cn; 2 Hunan Provincial Key Laboratory of the Traditional Chinese Medicine Agricultural Biogenomics, Changsha Medical University, Changsha 410219, China; 3 School of Medicine, Jishou University, Jishou 416000, China; weisiwen01@gmail.com; 4 Department of Physiology, School of Basic Medical Sciences, Hunan Normal University, Changsha 410013, China; wangrongrongzyk@163.com (R.W.); 202470193650@hunnu.edu.cn (Y.L.); 5 Key Laboratory of Model Animals and Stem Cell Biology in Hunan Province, Hunan Normal University School of Medicine, Changsha 410013, China

**Keywords:** *Acorus tatarinowii*, major depressive disorder, network pharmacology, molecular docking

## Abstract

Objective: To elucidate the molecular targets and mechanisms by which *Acorus tatarinowii* exerts therapeutic effects in major depressive disorder (MDD) using network pharmacology and molecular docking approaches. Methods: Bioactive compounds of *Acorus tatarinowii* were identified from comprehensive pharmacological databases. MDD-related targets were sourced from extensive genomic repositories. Overlapping targets were determined and subjected to network topology and protein–protein interaction (PPI) analyses to identify core targets. Gene Ontology (GO) and Kyoto Encyclopedia of Genes and Genomes (KEGG) pathway enrichment analyses were performed to reveal pertinent biological processes and signaling pathways. Molecular docking simulations validated the interactions between key bioactive compounds and core targets. Results: A total of 57 bioactive compounds were identified in *Acorus tatarinowii*, including apigenin, heterotropan, and isoelemicin. Integrative analysis revealed 700 compound-related targets and 2590 MDD-associated targets, with 150 intersecting targets. Network analyses pinpointed five core targets: TP53, STAT3, AKT1, PIK3CA, and PIK3R1. GO enrichment identified 858 significant biological processes, while KEGG pathway analysis highlighted 155 enriched pathways, notably the PI3K-Akt, cAMP, and MAPK signaling pathways. Molecular docking studies demonstrated strong binding affinities between key compounds and their respective targets. Conclusions: This study delineates the multifaceted polypharmacological mechanisms through which *Acorus tatarinowii* may confer protective effects against major depressive disorder, underscoring its potential as a promising therapeutic agent.

## 1. Introduction

Major depressive disorder (MDD), a debilitating psychiatric condition characterized by persistent affective disturbances (e.g., anhedonia and dysphoria), cognitive dysfunction (e.g., impaired attention and executive function), and psychomotor alterations, is frequently accompanied by somatic manifestations including sleep architecture disruption, appetite dysregulation, and maladaptive pain perception [1]. Epidemiological data from the World Health Organization (WHO) reveal that MDD affects over 300 million individuals globally, making it a leading contributor to disability−adjusted life years worldwide [2]. The clinical trajectory of MDD is often complicated by self-injurious behaviors and suicidal ideation, posing significant risks to both individual well-being and public health. Furthermore, the socioeconomic burden imposed by MDD, including healthcare costs and productivity losses, has been extensively documented. Current therapeutic paradigms predominantly rely on monoaminergic−based pharmacological interventions which are frequently limited by suboptimal response rates, treatment resistance, and a spectrum of adverse effects, including gastrointestinal disturbances, sleep disturbances, and sexual dysfunction [3,4]. In light of these limitations, the scientific community has witnessed a paradigm shift toward exploring alternative therapeutic strategies. Traditional Chinese medicine (TCM), with its multi−target mechanisms and historical documentation of neuropsychiatric applications, has emerged as a promising avenue for MDD treatment. This shift has catalyzed extensive research efforts to identify and characterize natural compounds with antidepressant potential, aiming to address unmet clinical needs in MDD management and improve therapeutic outcomes.

The rhizome of *Acorus tatarinowii* (ATR), a perennial herbaceous species within the Acoraceae family [5], has been pharmacologically characterized since its initial documentation in the classical Chinese medical text Shennong Bencao Jing. Mechanistic studies have elucidated that ATR exerts its therapeutic effects through multiple pathways, including orifice-opening, phlegm−resolving, and cognition−enhancing activities, as evidenced by both traditional applications and modern pharmacological investigations [6,7]. Clinically, ATR has been systematically employed in the management of a diverse array of disorders, spanning neurological, cardiovascular, gastrointestinal, and respiratory systems [8,9]. Its therapeutic scope extends to complex multifactorial conditions, including epilepsy, mood disorders (e.g., depression and anxiety), cognitive impairments (e.g., amnesia and dementia), altered states of consciousness, sleep disturbances (e.g., insomnia), communication disorders (e.g., aphasia), auditory dysfunction (e.g., tinnitus), oncological pathologies, cerebrovascular events (e.g., stroke), and dermatological diseases [10,11]. Emerging preclinical and clinical evidence posits that ATR may represent a promising therapeutic candidate for addressing unmet needs in neurodegenerative disorders (e.g., Alzheimer’s disease), psychiatric conditions (e.g., depression), and chronic inflammatory diseases (e.g., ulcerative colitis) [12,13]. The robust clinical efficacy of ATR, coupled with the ongoing discovery of its novel bioactive compounds and molecular mechanisms, has positioned it as a focal point of interdisciplinary research. This convergence of traditional knowledge and modern scientific inquiry underscores the potential of ATR as a source of innovative therapeutics, bridging the gap between traditional medicine and contemporary drug development paradigms.

Pharmacological investigations have demonstrated that the bioactive constituents of *Acorus tatarinowii* (ATR), notably β−asarone and α−asarone, exhibit a broad spectrum of therapeutic activities, including antioxidant, anti−inflammatory, and neuroprotective effects. These compounds have been further shown to modulate the release of key neurotransmitters, such as serotonin (5−HT) and dopamine (DA), which play critical roles in mood regulation and cognitive function. However, the therapeutic efficacy of ATR in major depressive disorder (MDD) is mediated by complex regulatory mechanisms involving multiple interconnected signaling pathways, rendering traditional pharmacological approaches insufficient for a comprehensive mechanistic understanding.

To address this complexity, network pharmacology has emerged as a powerful systems biology-based framework, enabling the systematic elucidation of multi−target and multi-pathway interactions between drug components, molecular targets, and disease mechanisms. This approach integrates predictive target identification, network topology analysis, and functional enrichment analysis to construct a holistic view of drug actions within biological systems.

In this study, we employ an integrated strategy combining network pharmacology with molecular docking techniques to systematically investigate the molecular mechanisms underlying the antidepressant effects of ATR [14]. By identifying key molecular targets and delineating the associated signaling pathways, this work aims to establish a mechanistic foundation for the clinical application of ATR and to advance the development of natural product-based therapeutics for MDD. This approach not only bridges the gap between traditional medicine and modern pharmacology but also provides a paradigm for elucidating the complex mechanisms of action of multi−component herbal therapies.

## 2. Materials and Methods

### 2.1. Identification of Active Compounds and Target Genes

The bioactive constituents of *Acorus tatarinowii* (ATR) were systematically identified and retrieved from the Traditional Chinese Medicine Systems Pharmacology Database (TCMSP; https://old.tcmsp-e.com/tcmsp.php, accessed on 14 January 2025), a comprehensive repository for traditional Chinese medicine compounds and their associated pharmacological properties. The screening criteria were established with thresholds of oral bioavailability (OB) ≥ 30% and drug−likeness (DL) ≥ 0.18 to systematically identify and prioritize the bioactive components of the herbal medicine. The Traditional Chinese Medicine Systems Pharmacology Database (TCMSP) utilizes a drug−likeness (DL) threshold of ≥0.18 to screen natural product derivatives for drug development potential. This threshold, derived from machine learning analysis of 2431 approved drugs, optimizes screening accuracy in natural product research [15]. Oral bioavailability (OB), the fraction of an orally administered compound which reaches systemic circulation, is critical for drug efficacy. Compounds with OB ≥ 30% typically achieve adequate absorption for therapeutic effects. Many phytochemicals show poor absorption due to structural complexity and low solubility, making the OB ≥ 30% standard valuable for identifying viable drug candidates [16]. Furthermore, the Herb Database (http://herb.ac.cn) was employed to complement and validate the initial findings. The active compounds were subsequently filtered in accordance with Lipinski’s rule of five to ensure drug-like properties. Structural representations of the compounds were obtained in SMILES format from the PubChem database (https://pubchem.ncbi.nlm.nih.gov, accessed on 14 January 2025); for compounds without available SMILES data in PubChem, MOL2 structure files were retrieved directly from the TCMSP database. This comprehensive approach ensured the robustness and reliability of the compound selection process. The molecular targets of the identified compounds were predicted using the Swiss Target Prediction platform (http://www.swisstargetprediction.ch/, accessed on 14 January 2025). Following the removal of duplicates and systematic organization, a refined set of key bioactive components and their corresponding target genes were obtained, providing a foundation for subsequent network pharmacology analysis.

### 2.2. Identification of Major Depressive Disorder-Related Targets and Venny Analysis

Using “Major depressive disorder” as the search term, human genes associated with this condition were systematically retrieved from the GeneCards database (https://www.genecards.org, accessed on 14 January 2025) and the OMIM database (https://www.omim.org, accessed on 14 January 2025). Following the removal of duplicate entries, a final set of target genes relevant to major depressive disorder was established. The identified drug targets were subsequently cross–referenced with the disease-related targets using Venny 2.1.0 (https://bioinfogp.cnb.csic.es/tools/venny, accessed on 14 January 2025), a Venn diagram tool, to identify overlapping targets which represent potential key nodes for therapeutic intervention.

### 2.3. Construction of Protein–Protein Interaction (PPI) Network

The protein–protein interaction (PPI) network was constructed and analyzed to identify core target interactions using the STRING database (https://cn.string-db.org, accessed on 14 January 2025). Common targets were imported into STRING, configuring the parameters to “multiple proteins”, selecting “Homo sapiens” as the species, and setting a confidence score threshold of ≥0.9. All other parameters were maintained at their default settings. The resulting PPI network data were subsequently analyzed in Cytoscape 3.10.3, employing the CytoNCA plugin to identify and prioritize core target proteins based on network topology metrics.

### 2.4. Network Construction and Analysis

The primary bioactive components of *Acorus tatarinowii* (ATR) and the overlapping disease targets were imported into Cytoscape 3.10.3 to construct a comprehensive network diagram. The “Analyze Network” function was utilized to perform topological analysis, enabling the identification and prioritization of key active components based on their network centrality and connectivity metrics.

### 2.5. GO Enrichment and KEGG Pathway Analysis

The intersecting target genes were imported into the DAVID database (https://davidbioinformatics.nih.gov, accessed on 14 January 2025) for biological information annotation, with “OFFICAL_GENE_SYMBOL” format selected and the species set to “Homo sapiens”. Enrichment analysis for Gene Ontology (GO) biological processes, cellular components, molecular functions (“GOTERM_BP_DIRECT”, “GOTERM_CC_ DIRECT”, “GOTERM_MF_DIRECT”), and KEGG pathways (“KEGG_PATHWAY”) was performed to elucidate the biological processes and key signaling pathways through which the drug exerts its effects on the disease. Results from the enrichment analysis were visualized using the Microbiome Analysis online platform (http://www.bioinformatics.com.cn, accessed on 14 January 2025).

### 2.6. Molecular Docking

The core active components of ATR were docked with their identified target proteins. The active compounds were downloaded in MOL2 format from the TCMSP database. These were processed using AutoDock Tools 1.5.6 and saved in “pdbqt” format to serve as ligands for molecular docking. Protein structures of core target proteins were obtained from the Protein Data Bank (PBD; https://www.rcsb.org, accessed on 14 January 2025). Water molecules and original ligands were removed using PyMOL version 3.1.0, and non-polar hydrogens and charges were added in AutoDock Tools 1.5.6 before being saved in “pdbqt” format as the docking receptor. AutoDock was employed for molecular docking by importing the ligands and receptors, configuring an appropriate docking box, and generating their respective conformations. Docking parameters were tailored to each target’s binding site characteristics, with grid boxes sized to ensure complete coverage of potential interaction regions. Detailed parameters for representative ligand–target pairs are available upon request. Conformations without hydrogen bonding were excluded, and the one with the lowest binding energy was selected as the optimal binding configuration. The docking results were visualized using PyMOL.

## 3. Results

### 3.1. Active Components of Acorus tatarinowii and Their Potential Targets

Using the TCMSP database, four active components of *Acorus tatarinowii* (ATR) were identified based on the criteria of oral bioavailability (OB) ≥ 30% and drug-likeness (DL) ≥ 0.18. Additionally, a search in the Herb Database yielded 54 active components, filtered according to Lipinski’s rule of five. After combining the results from both databases and removing duplicates, a total of 57 unique active components were retained. The PubChem Compound Identifier (CID) was utilized to obtain the isomeric SMILES representations of each candidate compound. For compounds lacking a PubChem CID, molecular depictions were downloaded from the TCMSP database, and their SMILES identifiers were calculated using the Structure Calculation platform (http://www.vcclab.org/web/alogps/, accessed on 14 January 2025). These resultant SMILES identifiers were then submitted to the Swiss Target Prediction database, leading to the identification of 700 potential targets for the active components.

### 3.2. Identification of Targets Related to Major Depressive Disorder (MDD)

**Figure 1:**
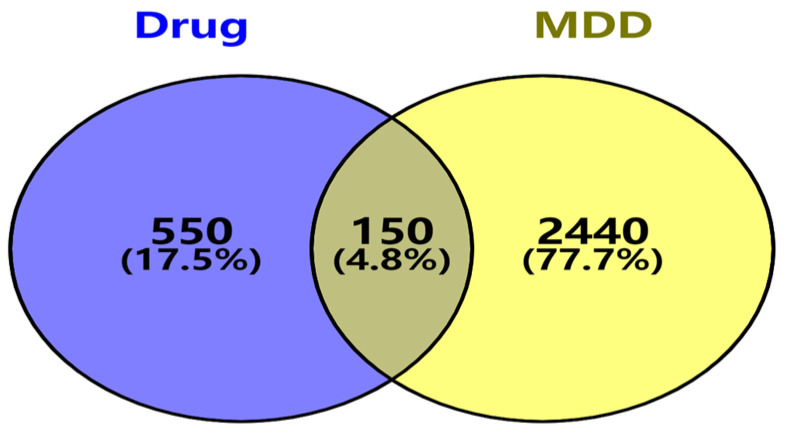
Intersection of *Acorus tatarinowii* and major depressive disorder (MDD) target points. (**Left**) (purple): 700 *Acorus tatarinowii* targets; (**right**) (yellow): 2590 MDD genes (GeneCards/OMIM). Intersection: 150 shared targets indicating potential therapeutic mechanisms (Venny 2.1.0).

A search using the keyword “Major depressive disorder” across the GeneCards and OMIM databases yielded a total of 16,230 relevant genes after deduplication. When relevance scores were applied to filter these genes, 1015 significant targets were selected. An additional 1776 targets were obtained from the OMIM database. By merging the gene lists from both databases and removing duplicates, a final set of 2590 genes associated with major depressive disorder was established. Subsequently, the drug targets identified earlier were compared with MDD related targets using the Venn diagram software, Venny 2.1.0. This analysis revealed a total of 150 common targets, indicating potential pharmacological interactions between the identified compounds and the disease (Figure 1).

### 3.3. Construction of the Potential-Target Protein–Protein Interaction (PPI) Network

**Figure 2:**
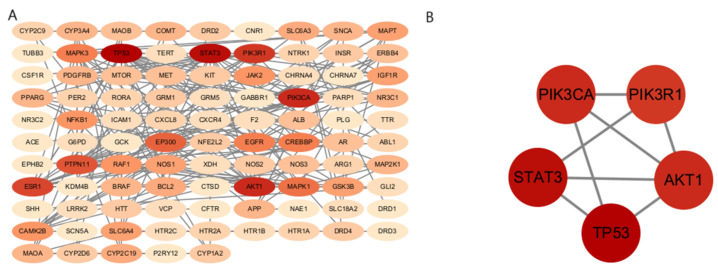
(**A**) PPI network of intersecting targets; (**B**) interaction map of core intersecting targets. Color Coding Explanation The gradient (light → dark) corresponds to the ranking score of core targets, where: Lighter colors: Targets with lower scores (peripheral nodes) Darker colors: Targets with higher scores (topologically central nodes) This scoring reflects each target’s network centrality based on topological analysis (e.g., degree/betweenness centrality).

The 150 identified potential targets were imported into the STRING database to construct a protei–protein interaction (PPI) network. This network was subsequently analyzed using Cytoscape 3.10.3 software, with the organism parameter set to “Homo sapiens” and a confidence score threshold of >0.9. The resulting PPI network comprised 150 nodes and 250 edges, representing a core interaction network of significant targets (Figure 2).

To delineate the core targets, a degre–based hierarchical analysis was performed on the connections between targets. The top-five core targets, characterized by high degree values, were identified as TP53, STAT3, AKT1, PIK3CA, and PIK3R1 (Figure 2).

### 3.4. Network of Active Components and Targets of Acorus tatarinowii

**Figure 3:**
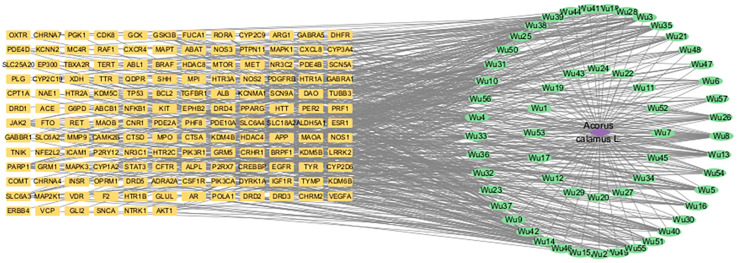
Network diagram of Traditional Chinese Medicine−component target interactions.

**Figure 4:**
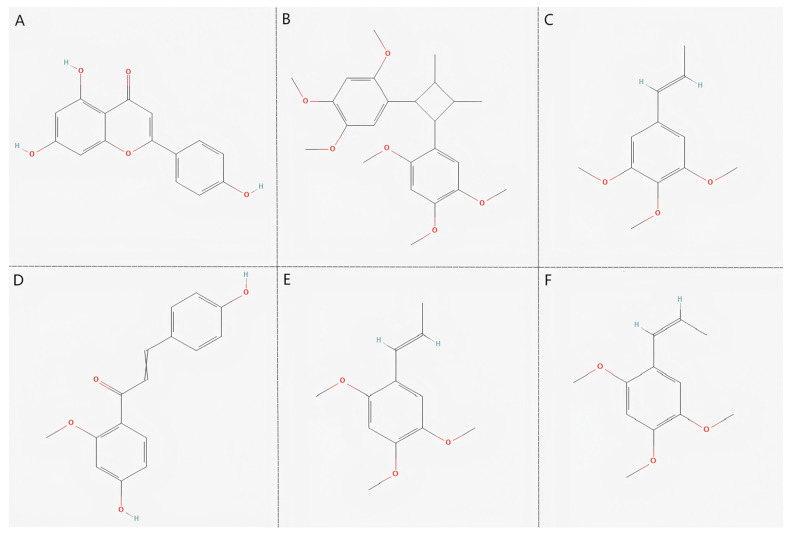
Chemical structures. (**A**) Apigenin; (**B**) heterotropan; (**C**) isoelemicin; (**D**) (1−(4−Hydroxy−2−methoxyphenyl)−3−(4−hydroxyphenyl)prop−2−en−1−one); (**E**) α−asarone; (**F**) β−asarone.

The active components of *Acorus tatarinowii* were mapped to their corresponding intersecting targets using Cytoscape 3.10.3, thereby constructing a “componen–target” network diagram (Figure 3). In this visualization, purple nodes represent traditional Chinese medicinal ingredients, green nodes denote active components, and yellow nodes signify targets. The density of connections among the nodes reflects their degree values, indicating their relative importance in the network. The key core components were determined by integrating multiple network metrics, including Betweenness Centrality, Closeness Centrality, Clustering Coefficient, and Degree (Table 1). The top six active components of *Acorus tatarinowii*, based on degree values, were identified as apigenin, heterotropan, isoelemicin, a chalcone derivative (1-(4-Hydroxy-2-methoxyphenyl)-3-(4-hydroxy-phenyl)prop-2-en-1-one), α-asarone, and β-asarone (Figure 4).

### 3.5. GO Enrichment Analysis and KEGG Pathway Enrichment Analysis

The Gene Ontology (GO) analysis identified 553 biological processes (BPs), 88 cellular component (CC) terms, and 217 molecular function (MF) terms. The top ten entries for each category were selected based on count and visualized as bar charts. The BP entries primarily relate to signal transduction, chromatin remodeling, and positive regulation of RNA polymerase II transcription. In terms of MF, the key functions involve protein binding, metal ion binding, and ATP binding. The CC entries predominantly include the plasma membrane, cytoplasm, and cell nucleus.

**Figure 5:**
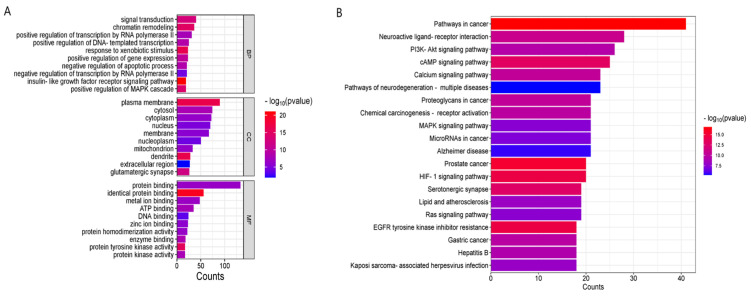
GO (**A**) and KEGG (**B**) pathway analysis profiles.

The KEGG pathway enrichment analysis revealed 155 pathways, which were ranked in descending order based on the number of enriched genes. The top 20 pathways were then depicted in bar charts, suggesting that *Acorus tatarinowii* may play significant roles in cancer pathways, neuroactive ligand–receptor interactions, the PI3K-Akt signaling pathway, the cAMP signaling pathway, and the calcium signaling pathway (Figure 5).

### 3.6. Molecular Docking

Molecular docking analysis was conducted to investigate the interactions between the primary active compounds of *Acorus tatarinowii* and its core target molecules, further elucidating the findings from network pharmacology. Effective components and targets with high degree values in the network were selected for docking to predict and score the potential therapeutic efficacy of *Acorus tatarinowii* in treating major depressive disorder (MDD). Based on the degree analysis, six key compounds were chosen as docking ligands: apigenin, heterotropan, isoelemicin, (1-(4-Hydroxy-2-methoxyphenyl)-3-(4-hydroxy-phenyl)prop-2-en-1-one), α-asarone, and β-asarone.

To assess the binding affinities of these constituents toward their respective targets, the top five core targets ranked by degree value were selected: TP53 (PDB ID: 8SVI), STAT3 (PDB ID: 6NJS), AKT1 (PDB ID: 8UW9), PIK3CA (PDB ID: 9ASF), and PIK3R1 (PDB ID: 6G6W). A negative binding energy indicates a spontaneous binding process, with values below −1.2 kcal/mol regarded as indicative of favorable docking outcomes.

**Figure 6:**
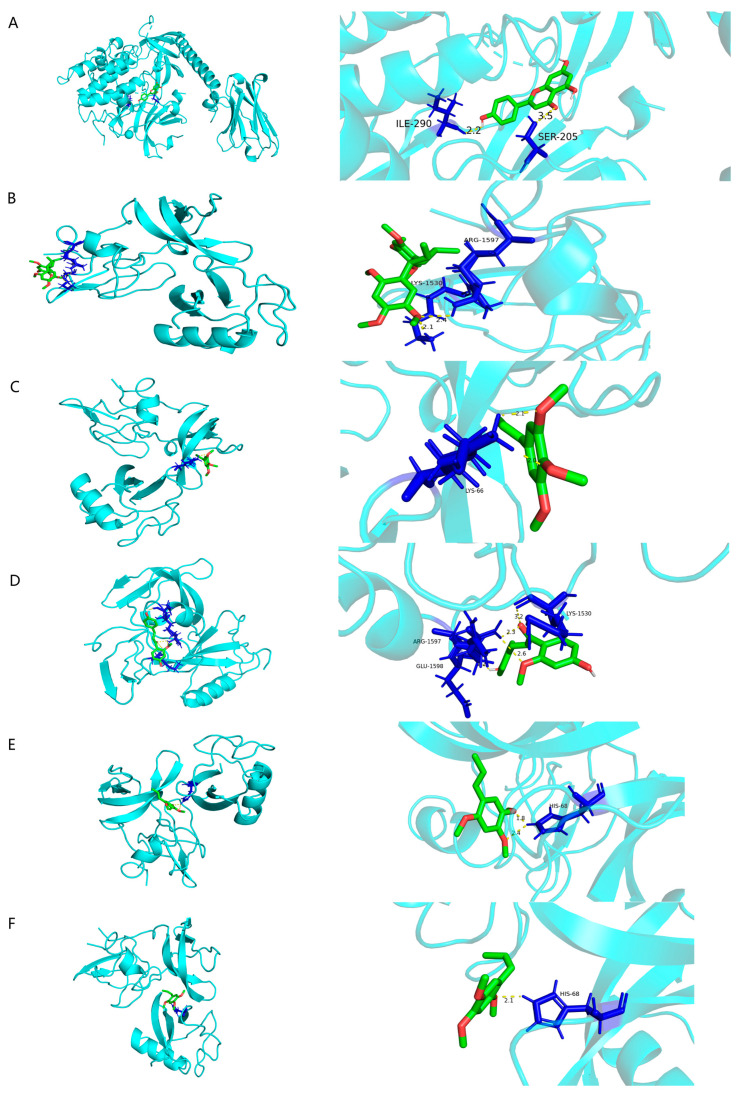
Molecular docking results showing key interactions. Yellow dashed lines represent hydrogen bonds Visualization of molecular docking results: (**A**) Apigenin with AKT1; (**B**) heterotropan with TP53; (**C**) isoelemicin with TP53; (**D**) (1-(4-Hydroxy-2-methoxyphenyl)-3-(4-hydroxy-phenyl)prop-2-en-1-one) with TP53; (**E**) α-asarone with TP53; (**F**) β-asarone with TP53.

Molecular docking simulations were visualized utilizing PyMOL, emphasizing the six core constituents that displayed the highest binding energies with potential targets (Figure 6). The observed hydrogen bonds formed between the ligands and receptors signify stable interactions. The results of the molecular docking analysis demonstrated a strong affinity between the identified compounds and their corresponding targets (Table 2).

## 4. Discussion

The pathogenesis of major depressive disorder (MDD) has been increasingly elucidated as a multifactorial process involving interconnected biological and psychosocial mechanisms. Central to this understanding are several wel–established hypotheses, including dysregulation of the hypothalamic–pituitary–adrenal (HPA) axis [17], monoaminergic system dysfunction, neuroinflammatory processes, genetic and epigenetic modifications, neuroanatomical and functional plasticity, and the influence of psychosocial stressors [18,19]. Within this complex etiological framework, *Acorus tatarinowii*, a pharmacologically significant herb in traditional Chinese medicine, has been identified as a promising therapeutic candidate for MDD [20]. Phytochemical profiling of *Acorus tatarinowii* has revealed a diverse array of bioactive constituents, with over 160 distinct compounds identified, including phenylpropanoids, terpenoids, lignans, flavonoids, alkaloids, amides, and organic acids. Pharmacological investigations have demonstrated that these compounds exhibit a broad spectrum of bioactivities, such as antidepressant, anticonvulsant, anxiolytic, anti-fatigue, and antifungal properties. Notably, *Acorus tatarinowii* has been shown to exert potent neuroprotective effects [9] which are mediated through the modulation of neurotransmitter systems and enhancement of cerebral blood flow, thereby attenuating neuronal injury. Its efficacy in protecting the central nervous system has been validated both as a monotherapy and in combination with other pharmacological agents. This accumulating body of evidence underscores the therapeutic potential of *Acorus tatarinowii* in addressing the multifactorial nature of MDD. Further investigation within preclinical and clinical frameworks is warranted to fully elucidate its mechanisms of action and optimize its therapeutic applications. Such efforts may provide novel insights into the development of multi-target strategies for the treatment of complex neuropsychiatric disorders.

This study employed an integrated approach combining network pharmacology and molecular docking to systematically elucidate the active compounds, molecular targets, and signaling pathways underlying the therapeutic effects of *Acorus tatarinowii* in major depressive disorder (MDD). Network analysis identified several key bioactive constituents, including apigenin, heterotropan, isoelemicin, a chalcone derivative (1-(4-hydroxy-2-methoxyphenyl)-3-(4-hydroxyphenyl)prop-2-en-1-one), α-asarone, and β-asarone, which collectively contribute to its pharmacological profile. Apigenin, a naturally occurring flavonoid with widespread distribution in plants, has been demonstrated to exhibit a broad spectrum of bioactivities, including anti-apoptotic, anti-inflammatory, and antioxidant properties [21]. Mechanistic studies have revealed that apigenin inhibits the COX-2 and NF-κB pathways, reduces reactive oxygen species (ROS) generation, activates the PI3K-AKT signaling cascade, and upregulates brain-derived neurotrophic factor (BDNF) expression, thereby enhancing synaptic plasticity and conferring neuroprotection [22,23]. Additionally, apigenin has been shown to induce apoptosis in tumor cells through suppression of the MAPK/ERK and PI3K-AKT pathways, underscoring its dual role in neuroprotection and oncological contexts [24]. Heterotropan, a natural lignan isolated from plants of the Asarum genus [25], has been reported to inhibit the release of pro-inflammatory cytokines, such as TNF-α and IL-6, likely via blockade of the NF-κB signaling pathway. Other constituents, including isoelemicin, chalcone derivatives, α-asarone, and β-asarone, have also been demonstrated to exhibit significant anti-inflammatory and antioxidant activities, further contributing to the multifaceted pharmacological effects of *Acorus tatarinowii*. Collectively, these findings posit that *Acorus tatarinowii* exerts its therapeutic potential in MDD through a multi-target mechanism of action, encompassing modulation of inflammatory pathways, reduction in oxidative stress, and enhancement of neuroplasticity. This integrated pharmacological profile highlights *Acorus tatarinowii* as a promising candidate for further preclinical and clinical investigation, offering a potential avenue for the development of novel therapeutics for complex neuropsychiatric disorders [26].

Protein–protein interaction (PPI) network analyses have identified several core molecular targets through which the active compounds of *Acorus tatarinowii* may exert their therapeutic effects. These compounds include TP53, STAT3, AKT1, PIK3CA, and PIK3R1. TP53, encoding the p53 protein, functions as a transcription factor that responds to cellular stressors such as DNA damage and oxidative stress, regulating critical biological processes including cell cycle control, apoptosis, metabolic homeostasis, and genomic stability [27,28]. Within the nervous system, TP53 plays a pivotal role in neuronal survival, synaptic plasticity, and the modulation of neuroinflammatory responses. Emerging evidence suggests that TP53 influences neuronal survival and synaptic plasticity, in part, through the regulation of brain-derived neurotrophic factor (BDNF) expression [29]. Additionally, TP53 has been shown to confer neuroprotective effects against oxidative stress, a mechanism implicated in the pathophysiology of major depressive disorder (MDD) [30]. Furthermore, TP53 modulates the expression of pro-inflammatory cytokines, thereby influencing neuroinflammation and depressive behaviors [31]. These findings highlight the multifaceted role of TP53 in both neuroprotection and the regulation of neuroinflammatory processes, underscoring its potential as a critical target in the therapeutic mechanisms of *Acorus tatarinowii* for MDD.

STAT3, a transcription factor central to diverse biological processes including cell proliferation, differentiation, apoptosis, and immune regulation [32], has been implicated in the pathophysiology of major depressive disorder (MDD). Clinical evidence demonstrates that STAT3 levels are significantly elevated in patients with MDD, suggesting its potential involvement in disease mechanisms [33]. STAT3 plays a critical role in mediating inflammatory responses and is posited to contribute to MDD pathogenesis through its regulatory effects on microglial and astrocytic functions [34]. Pro-inflammatory cytokines, such as IL-6, have been shown to activate STAT3 via the JAK-STAT signaling pathway, a process linked to the induction of neuroinflammation and the manifestation of depressive symptoms [35]. Furthermore, MDD is frequently characterized by dysregulation of the hypothalamic–pituitary–adrenal (HPA) axis, a key neuroendocrine stress response system. STAT3 has been demonstrated to modulate HPA axis activity, thereby influencing stress responsiveness and depressive behaviors. These findings collectively highlight the dual role of STAT3 in neuroinflammatory processes and neuroendocrine regulation, underscoring its significance as a molecular target in the pathophysiology and potential therapeutic intervention of MDD.

AKT1, a serine/threonine kinase central to critical biological processes such as cell survival, proliferation, metabolism, and growth [36], has been implicated in the pathophysiology of major depressive disorder (MDD) [37]. Mechanistic studies have elucidated that AKT1 contributes to MDD through its regulation of neuroplasticity, modulation of neuroinflammatory pathways, and its influence on hypothalamic–pituitary–adrenal (HPA) axis activity [38]. Specifically, AKT1 signaling has been demonstrated to play a pivotal role in maintaining synaptic plasticity and promoting neuronal survival, while its dysregulation is associated with heightened neuroinflammation and HPA axis hyperactivity, both of which are hallmark features of MDD. These findings underscore the multifaceted role of AKT1 in the neurobiological mechanisms underlying MDD, highlighting its potential as a therapeutic target for intervention.

PIK3CA and PIK3R1, critical regulatory components of the PI3K-AKT-mTOR signaling pathway, play essential roles in modulating neuroplasticity, neuroinflammation, and metabolic homeostasis, all of which are implicated in the pathological mechanisms of major depressive disorder (MDD) [39,40]. Specifically, these proteins contribute to the regulation of synaptic plasticity, neuronal survival, and inflammatory responses, processes that are frequently dysregulated in MDD. In summary, the active components of *Acorus tatarinowii* are posited to exert their therapeutic effects on MDD through interactions with these pivotal molecular targets. By modulating cellular functions and signaling pathways associated with neuronal health and inflammatory responses, *Acorus tatarinowii* may address the multifactorial nature of MDD, offering a potential avenue for the development of novel therapeutic strategies.

Gene Ontology (GO) enrichment analysis revealed that biological processes (BPs) were predominantly associated with signal transduction, chromatin remodeling, and positive regulation of RNA polymerase II transcription. Molecular functions (MFs) were primarily linked to protein binding, metal ion binding, and ATP binding, while cellular components (CC) were predominantly localized to the plasma membrane, cytoplasm, and nucleus.

KEGG pathway analysis identified several pathways implicated in the pathophysiology of major depressive disorder (MDD), including cancer pathways, neuroactive ligand–receptor interactions, the PI3K-Akt signaling pathway, the cAMP signaling pathway, the calcium signaling pathway, neurodegenerative disease pathways, proteoglycans in cancer, chemical carcinogenesis-receptor activation, the MAPK signaling pathway, and the Alzheimer’s disease signaling pathway. Notably, the PI3K-Akt, cAMP, and MAPK signaling pathways were found to be significantly associated with MDD.

The PI3K-AKT signaling pathway, a critical regulator of cell survival, proliferation, metabolism, and neuroplasticity, has been demonstrated to modulate neuronal survival and synaptic plasticity through downstream effectors such as mTOR and GSK-3β [41]. Reduced AKT activity, frequently observed in MDD, has been linked to neuronal atrophy in the hippocampus and prefrontal cortex, impairing synaptic function by inhibiting the synthesis of synaptic proteins (e.g., PSD-95, Synapsin). Preclinical studies have demonstrated that AKT knockout mice exhibit depressive-like behaviors, while clinical studies have reported reduced AKT phosphorylation levels in the peripheral blood of MDD patients [42].

The cAMP signaling pathway, which regulates cellular processes such as proliferation, differentiation, and apoptosis [43,44], is activated by extracellular ligands binding to G protein-coupled receptors (GPCRs), leading to the dissociation of the Gα subunit and subsequent activation of adenylyl cyclase (AC). This cascade catalyzes the conversion of ATP to cAMP, which promotes the phosphorylation of protein kinase A (PKA) and activation of cAMP response element-binding protein (CREB), ultimately upregulating BDNF expression [45,46]. BDNF deficiency, a hallmark of MDD, is directly associated with reduced hippocampal neurogenesis [47]. Dysregulation of monoamine neurotransmitters, such as serotonin (5-HT) and norepinephrine (NE), which activate cAMP signaling through GPCRs (e.g., 5-HT1A, β-AR), may contribute to reduced cAMP levels in MDD.

The MAPK signaling pathway, which regulates cell proliferation, differentiation, and apoptosis, is also implicated in MDD. Extracellular signal-regulated kinase (ERK) promotes synaptic plasticity and neurogenesis by modulating transcription factors such as Elk-1. Chronic stress has been shown to inhibit ERK phosphorylation, leading to hippocampal neuronal atrophy. Inflammatory cytokines, including IL-6 and TNF-α, activate p38 MAPK, inducing microglial activation and neurotoxic responses, thereby supporting the neuroinflammatory hypothesis of MDD. Additionally, excessive activation of JNK signaling has been linked to neuronal apoptosis, particularly in chronic unpredictable stress (CUS) models. The interplay between these pathways underscores the complexity of MDD pathogenesis and highlights the need for multifaceted therapeutic strategies targeting multiple underlying mechanisms.

In summary, the bioactive constituents of *Acorus tatarinowii* may exert effects on MDD through key targets such as TP53, STAT3, AKT1, PIK3CA, and PIK3R1, thereby implicating the PI3K/AKT, cAMP, and MAPK signaling pathways. Enrichment analyses, including Gene Ontology (GO) and Kyoto Encyclopedia of Genes and Genomes (KEGG), elucidated the involvement of these targets in anti-inflammatory processes, regulation of cell proliferation and apoptosis, and immunomodulation, highlighting the significance of the PI3K-AKT signaling pathway in the context of depression.

This study posits that the active components of *Acorus tatarinowii* act directly on MDD. However, it remains to be determined whether other constituents of the extract may exert therapeutic effects through indirect mechanisms. Furthermore, future research should investigate whether varying concentrations of these bioactive components influence the efficacy in treating MDD.

## 5. Conclusions

In this study, we employed a network pharmacology approach to systematically elucidate the active components, key molecular targets, and signaling pathways underlying the therapeutic effects of *Acorus tatarinowii* in major depressive disorder (MDD). Molecular docking analyses further validated the interactions between core bioactive constituents and pivotal disease-related targets. Our findings suggest that the mechanism of action of *Acorus tatarinowii* in MDD treatment is characterized by a multi-component, multi-target, and multi-pathway model, reflecting the complex pathophysiology of the disorder. However, these conclusions require further experimental validation through in vitro and in vivo studies, as well as clinical investigations, to substantiate the proposed mechanisms and establish their translational relevance.

## Figures and Tables

**Table 1:** Topological properties of candidate hub nodes in the protein interaction network.

Node	Average Shortest Path Length	Betweenness Centrality	Closeness Centrality	Clustering Coefficient	Degree	Eccentricity
Apigenin	2.469	0.0482	0.405	0	27	3
Heterotropan	2.469	0.0852	0.405	0	27	3
Isoelemicin	2.469	0.0470	0.405	0	27	3
1-(4-Hydroxy-2-methoxyphenyl)-3-(4-hydroxyphenyl)prop-2-en-1-one	2.478	0.0977	0.404	0	26	3
Alpha-asarone	2.488	0.0299	0.402	0	25	3
Beta-asarone	2.488	0.0308	0.402	0	25	3

**Table 2:** Binding affinity values of key constituents of *Acorus tatarinowii* with core targets.

Potential Core Targets		Binding Affinity Values (kcal/mol)					
Target	PDB ID	Apigenin	Heterotropan	Isoelemicin	(1-(4-Hydroxy-2-methoxyphenyl)-3-(4-hydroxy-phenyl)prop-2-en-1-one)	α-Asarone	β-Asarone
TP53	8SVI	−5.07	−3.95	−3.78	−4.68	−4.35	−4.21
STAT3	6NJS	−3.96	−1.98	−3.14	−3.3	−2.38	−2.65
AKT1	8UW9	−6.34	−2.91	−3.3	−3.61	−4.17	−3.12
PIK3CA	9ASF	−5.34	−2.91	−2.94	−4.06	−3.29	−3.03
PIK3R1	6G6W	−2.83	−1.89	−2.46	−2.23	−2.29	−2.19

## Data Availability

The original contributions presented in this study are included in the article. Further inquiries can be directed to the corresponding authors.
